# Metabolomic investigation of regional brain tissue dysfunctions induced by global cerebral ischemia

**DOI:** 10.1186/s12868-016-0256-9

**Published:** 2016-05-20

**Authors:** Tianshu Zhang, Wei Wang, Jin Huang, Xia Liu, Haiyan Zhang, Naixia Zhang

**Affiliations:** Department of Analytical Chemistry, Shanghai Institute of Materia Medica, Chinese Academy of Sciences, Shanghai, 201203 China; CAS Key Laboratory of Receptor Research, Shanghai Institute of Materia Medica, Chinese Academy of Sciences, Shanghai, 201203 China; Shanghai Key Laboratory of New Drug Design, School of Pharmacy, East China University of Science and Technology, Shanghai, 200237 China

**Keywords:** Bilateral common carotid arteries occlusion, Metabolomics, NMR, Brain tissue dysfunctions, Amino acid metabolism

## Abstract

**Background:**

To get a broader view of global ischemia-induced cerebral disorders at the metabolic level, a nuclear magnetic resonance-based metabolomic study was performed to evaluate the metabolic profile changes on regional brain tissues of female and male mice upon bilateral common carotid arteries occlusion (BCCAO) operation.

**Results:**

Significant metabolic disorders were observed in both cerebral cortex and hippocampus tissues of the experimental mice upon global cerebral ischemic attack. Multiple amino acids were identified as the dominantly perturbed metabolites. It was also shown that although the metabolic profile change patterns in the brain tissues were quite similar in male and female BCCAO mice, metabolic disorders in the cortex tissues were more severe in the female mice than in the male mice.

**Conclusions:**

In the present study, significant changes in amino acid metabolic pathways were confirmed in the early stage of global ischemia. Meanwhile, cerebral metabolic dysfunctions were more severe in the female BCCAO mice than in the male mice, suggesting that gender may play a role in different metabolic responses to the ischemic attack, which may provide an important hypothesis for a better understanding of the clinically observed gender-dependent pathological outcome of cerebral ischemia.

**Electronic supplementary material:**

The online version of this article (doi:10.1186/s12868-016-0256-9) contains supplementary material, which is available to authorized users.

## Background

Ischemic stroke, which is usually initiated by the accidental occurrence of thrombosis, is one of the most common causes of death in the population over 65 years old. Endothelial dysfunction, neurovascular crosstalk perturbation, and blood brain barrier (BBB) disruption have been identified to be the risk factors for blood vessel occlusion/ischemic stroke [1–3]. After the occurrence of cerebral thrombosis, focal brain ischemia develops in the brain tissues and then leads to a series of cellular disorders and metabolic disturbances including mitochondrial dysfunction [4], oxidative stress, NO deficit [5], inflammation [6], increased anaerobic glycolysis [7, 8], aberrant turnover of protein synthesis [9], and dysfunction of glutamate-glutamine cycle [10] etc. All the above-mentioned cellular changes link to a final irreversible damage in the brain tissues. The underlying molecular mechanisms of ischemic stroke are quite complicated, and are not yet fully understood. Emerging systematic research approaches, such as genomics, proteomics, and metabolomics, provide new opportunities for getting a broader view of ischemia-induced cerebral disorders.

Genomic and proteomic studies can detect the changes that happened on gene and protein level, while metabolomics can reveal whole metabolic profile changes of living systems in response to external stimuli such as disease-induced damage and drug treatments by monitoring the endogenous low molecular weight metabolites [11–15]. Metabolomics has proven to be valuable in the investigation of molecular mechanisms underlying various human diseases [16, 17]. In the present study, a nuclear magnetic resonance (NMR)-based metabolomic study, which has not ever been tried for the bilateral common carotid artery occlusion (BCCAO) ischemic mouse model, was performed to investigate the biochemical mechanisms of cerebral disorders happening in the early stage of global ischemic attack/stroke.

In Wang et al.’s study, transient ischemia induced by BCCAO procedure has been demonstrated to cause cerebral functional changes (memory deficits) in experimental mice. In the present study, the same BCCAO procedure was conducted in the experimental mice group to induce ischemic defects. Meanwhile, the sham mice group was subjected to the sham procedure by following the same surgical procedure without artery occlusion. The cortex and hippocampus tissues of BCCAO and sham-operation mice were obtained and subjected to NMR analysis 1 h after the surgery to evaluate the functional state of regional brain tissues in the early stage of ischemic attack. Multivariate analysis of principal component analysis (PCA), partial least squares discriminant analysis (PLS-DA), and orthogonal partial least squares discriminant analysis (OPLS-DA) were conducted to evaluate possible correlations between the metabolic profile changes and the variations in biological pathways in the BCCAO mice.

## Results

### ^1^H NMR spectra of cerebral tissue samples

**Fig. 1:**
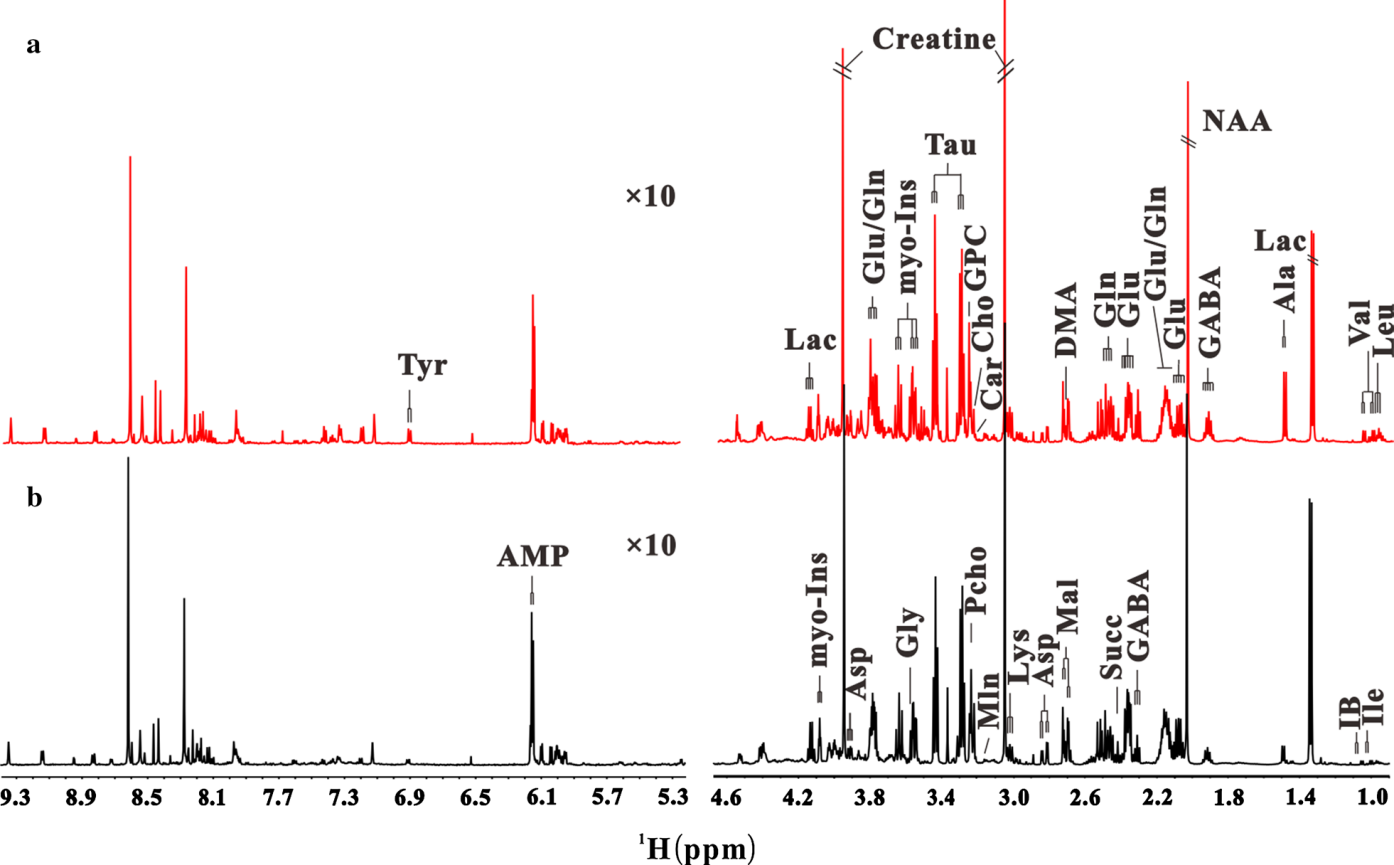
The 600-MHz ^1^H NMR NOESY spectra (δ 0.9–4.7, 5.3–9.4) of aqueous extracts from the cortex tissues of mice in female experimental group (**a**) and female sham group (**b**). The abbreviations of metabolites were shown in Additional file 1: Table S1

Representatives of two 1D ^1^H NMR spectra of the cortex tissue aqueous extracts obtained from the experimental and sham group mice were shown in Fig. 1, and 1D ^1^H NMR spectra of hippocampus tissue samples were illustrated in Additional file 1: Figure S1. The assignments (both chemical shift and multiplicity) of identified metabolites (Additional file 1: Table S1) were based on reported results [18, 19] and the Human Metabolome Database (HMDB). The NMR spectra of cortex tissue samples contained 26 assignable metabolites (Additional file 1: Tables S2, S3; Fig. 1), while the hippocampus tissue samples contained 25 assignable metabolites (Additional file 1: Tables S2, S3, Figure S1). The identified metabolites include amino acids (leucine, isoleucine, valine, alanine, lysine, glutamine, glutamate, aspartate, *N*-acetylaspartate (NAA) and tyrosine, γ-aminobutryric acid (GABA), glycine), carboxylic acid (succinate, malate, lactate, isobutyrate, malonate, creatine), membrane component (choline, sn-glycero-3-phophocholine (GPC), o-phosphocholine), oxidative stress-related metabolites (taurine), and nitrogen-containing heterocyclic molecules (adenosine monophosphate (AMP), dimethylamine (DMA), carnitine and myo-inositol).

### Metabolic disorders in cerebral cortex of BCCAO mice

**Fig. 2:**
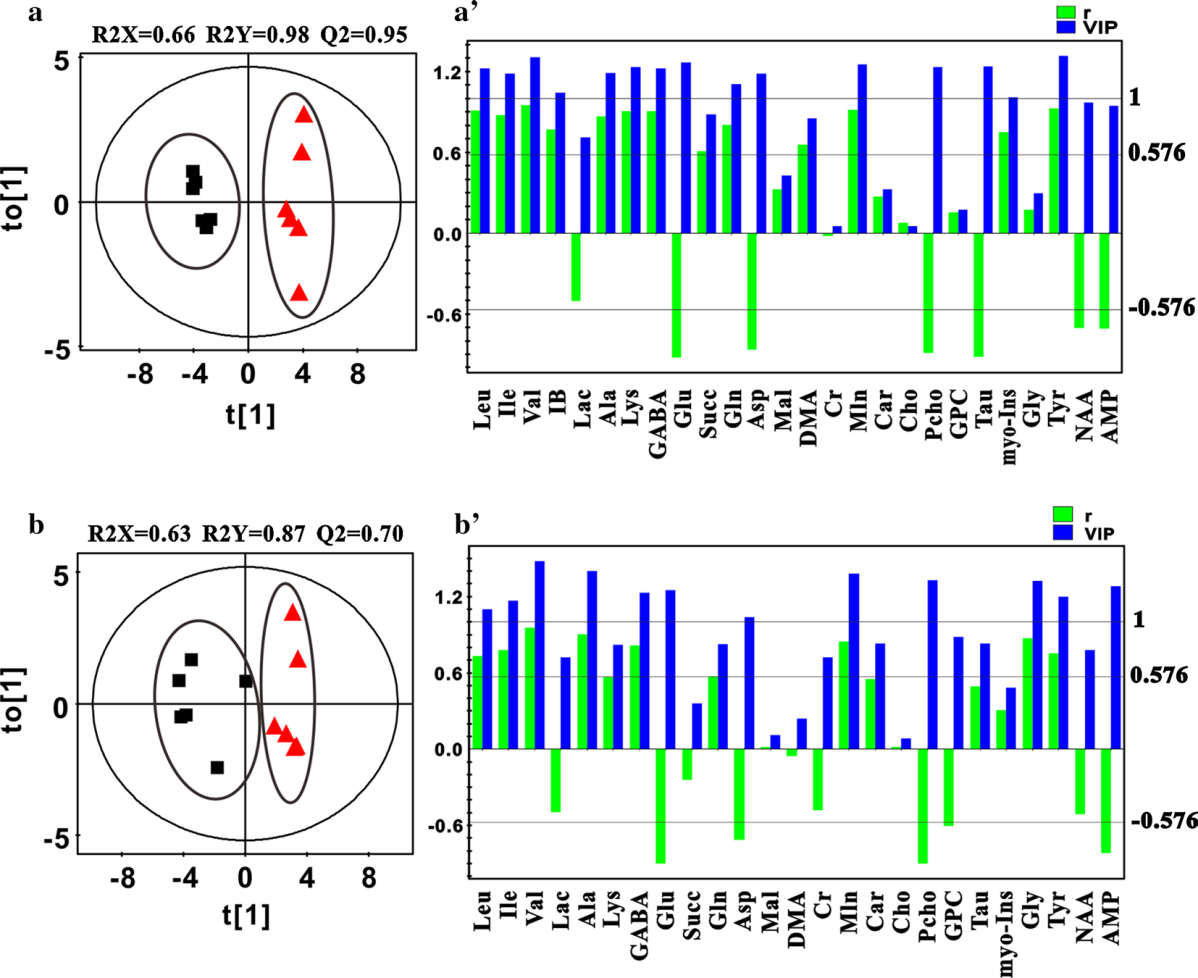
The OPLS-DA score plots and loading plots derived from the 600 MHz ^1^H NMR spectra of cortex samples (**a**, **a**′) and hippocampus samples (**b**, **b**′) extracted from the mice in female sham group (*filled square*) and female experimental group (*triangle*). Each group contains six samples (n = 6). The values of Q2 parameter, which were > 0.4, indicated that the established OPLS-DA models were valid. Those metabolites with the value of variable importance in the projection (VIP) bigger than 1 and the absolute value of correlation coefficient |r| greater than the cutoff value of 0.576 were defined to be the discriminative metabolites for group clustering. The discriminative metabolites with the value of correlation coefficient r > 0.576 indicated that their levels increased upon the global ischemic attack. And those discriminative metabolites with the value of correlation coefficient r < −0.576 indicated that their levels decreased upon the global ischemic attack

**Fig. 3:**
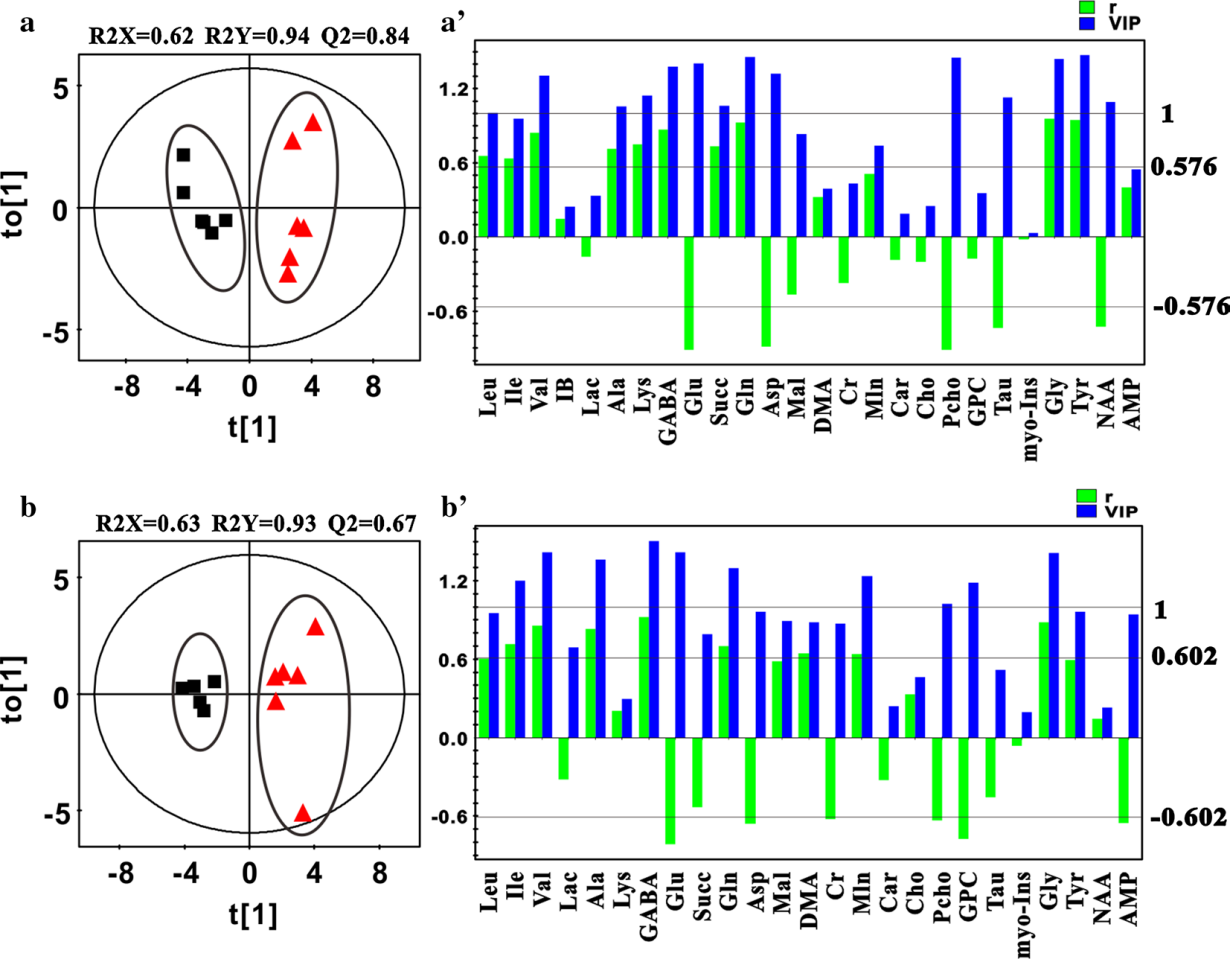
The OPLS-DA score plots and loading plots derived from the 600 MHz ^1^H NMR spectra of cortex samples (**a**, **a**′) and hippocampus samples (**b**, **b**′) extracted from the mice in male sham group (*filled square*) and male experimental group (*triangle*). The male hippocampus sham group contains five samples (n = 5), and the other three groups contain six samples (n = 6) in each. The values of Q2 parameter, which were >0.4, indicated that the established OPLS-DA models were valid. Those metabolites with the value of variable importance in the projection (VIP) bigger than 1 and the absolute value of correlation coefficient |r| greater than the cutoff value 0.576 (the sum of two group samples equals to 12, n = 12) or 0.602 (the sum of two group samples equals to 11, n = 11) were defined to be the discriminative metabolites for group clustering. The discriminative metabolites with the value of correlation coefficient r > 0.576 (n = 12) or r > 0.602 (n = 11) indicated that their levels increased upon the global ischemic attack. And those discriminative metabolites with the value of correlation coefficient r < −0.576 (n = 12) or r < −0.602 (n = 11) indicated that their levels decreased upon the global ischemic attack

The PCA (Additional file 1: Figure S2, Figure S3), PLS-DA (Additional file 1: Figure S2, Figure S3) and OPLS-DA (Figs. 2, 3) score plots revealed clear separations between the cortex samples from female/male experimental group and the sham group. The permutation test data (Additional file 1: Figure S2, Figure S3), in which all of the permutated Q2 values to the left were lower than the original data point to the right, indicating that the original PLS-DA and OPLS-DA models were valid. Based on the determined coefficient numbers of (|r|) and VIP from the back-transformed OPLS-DA coefficient plots and the *p* values from the student’s *t* tests, the major discriminative metabolites for each group were indentified in the pair-wised comparison (Figs. 2, 3, Additional file 1: Tables S2, S3). Upon BCCAO operation, increased levels of leucine, isoleucine, valine, alanine, lysine, GABA, succinate, glutamine and tyrosine and decreased levels of glutamate, aspartate, o-phosphocholine, taurine and NAA were observed in both the female and male cortex tissues. Additional metabolite perturbations including the up-regulations of isobutyrate, DMA, malonate, myo-inositol, and the down-regulation of AMP were detected in the female cortex samples, suggesting that more severe metabolic damage was induced by global cerebral ischemia/reperfusion in the female cortex region.

### Metabolic disorders in hippocampus of BCCAO mice

Distinct separations between the hippocampus samples from the female/male experimental group and the sham group were revealed by PCA (Additional file 1: Figure S2, Figure S3), PLS-DA (Additional file 1: Figure S2, Figure S3) and OPLS-DA (Figs. 2, 3) score plots. The validation plots obtained by permutation tests with 350 permutated models from the first component (Additional file 1: Figure S2, Figure S3) indicated that the original PLS-DA and OPLS-DA models were valid. According to the determined coefficient values of VIP and |r| (Figs. 2, 3, Additional file 1: Tables S2, S3) and the *p* values, the major discriminative metabolites for the hippocampus groups were identified. Upon BCCAO procedure, the levels of isoleucine, valine, alanine, GABA, malonate and glycine were up regulated and the levels of glutamate and o-phosphocholine were down regulated in both the female and male hippocampus tissues. Meanwhile, the increased levels of leucine and tyrosine and the decreased levels of aspartate and AMP were observed in the female hippocampus samples only, and the increased level of glutamine and the decreased level of GPC were only detected in the male hippocampus tissues.

### Ca^2+^-induced brain mitochondrial swelling

Ca^2+^ exposure could induce significant swelling of the isolated mitochondria. The swelling level is estimated by changes in light scattering at 540 nm as monitored with a microplate reader. A decrease in absorbance indicates an increase in mitochondrial swelling, and a faster decrease in absorbance during experimental time scale is usually caused by mitochondria dysfunction. The decrease slope of the absorbance at 540 nm from cycle 1 to cycle 30 was calculated with linear regression to represent mitochondrial swelling degree. In our study, in comparison with the female/male sham group, the Ca^2+^-induced mitochondrial swelling in the cortex tissues of female/male BCCAO group mice exhibited a much more rapid decrease in swelling slope (Additional file 1: Figure S4A) and in absorbance during the 30-min experimental duration (Additional file 1: Figure S4B). Although the changes in mitochondrial swelling are statistically significant, the mild increase suggests that the BCCAO operation induced slight mitochondria dysfunction of the cortex tissues of experimental mice.

## Discussion

To get a broader view of the biochemical mechanisms of ischemia-induced cerebral disorders, we performed a holistic evaluation of the metabolic profile perturbations that occurred in regional mouse brain tissues upon BCCAO operation. The metabolomics data showed that global ischemia/reperfusion induced significant metabolic disorders in the cerebral cortex and hippocampus tissues of the experimental mice. In the meantime, it also demonstrated that upon ischemia/reperfusion operation a dominant number of perturbed metabolites in the cerebral cortex and hippocampus of experimental mice were amino acids. These amino acids functioned as energy providing materials and/or functional components in brain tissues (Additional file 1: Tables S2, S3). Besides, it is worth noting that the female BCCAO mice showed more severe metabolic disorders in their cortex tissues than the male mice (Additional file 1: Tables S2, S3).

### Potential biochemical mechanisms related to ischemia-induced cerebral disorders of female mice

As it has been mentioned above, significant metabolic disorders were observed in the cerebral cortex and hippocampus tissues of female BCCAO mice. Clear group clustering for the female experimental group and the female sham group were revealed by the OPLS-DA score plots (Fig. 2). Besides, it was clearly shown by the metabolomics analysis data that the induced metabolic dysfunctions in regional brain tissues of female mice focused on energy metabolism, amino acid metabolism, oxidative stress, and cell state-related pathway.

**Fig. 4:**
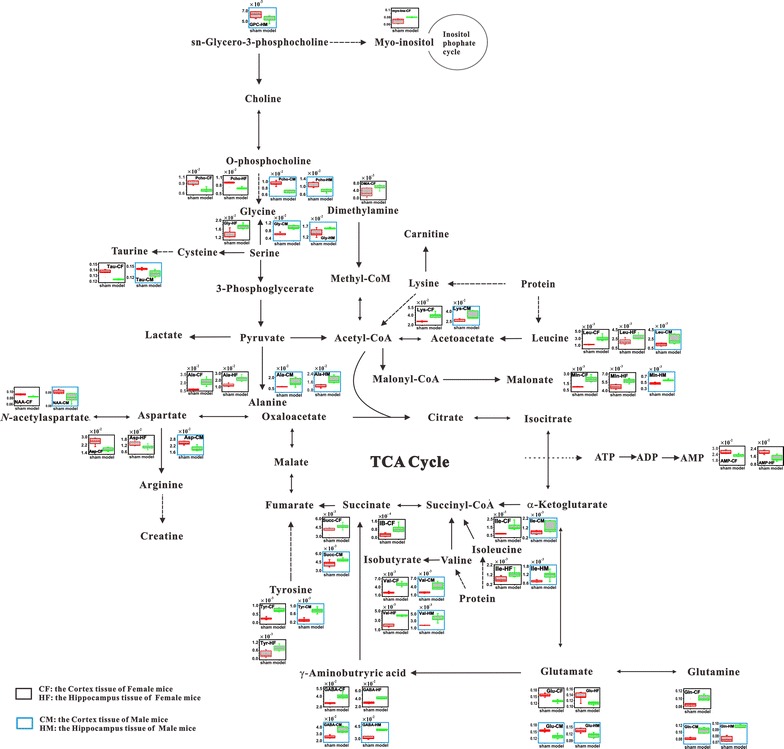
The potential metabolic pathways disturbed by ischemia in the regional brain tissues of female and male experimental group mice

After the BCCAO procedure, the level of AMP dramatically decreased in the cortex and hippocampus tissues of female mice (Fig. 4), indicating insufficient energy supply in these tissues. Consistent with this observation, the levels of some well-known energy-providing amino acids, including leucine, isoleucine, valine, tyrosine and lysine, increased significantly in brain tissues of female BCCAO mice, indicating that protein breaking-down was up-regulated (Fig. 4) [11, 20, 21]. Furthermore, down-regulations were observed for glutamate and aspartate upon ischemia/reperfusion damaging. Since glutamate and aspartate could be directly transformed to tricarboxylic acid cycle (TCA cycle) intermediate 2-oxoglutaramate and oxaloacetate, respectively, we believe that the acute depletions of these two amino acids in the cortex and hippocampus tissues of female mice might be due to their remediation and compensation in the energy-providing TCA cycle [22, 23]. This hypothesis is also supported by the results that mitochondria dysfunction in the cerebral cortex cells of female BCCAO mice was somehow more severe than that of the female mice from the sham group (Additional file 1: Figure S4). The changes of above-mentioned energy-related metabolites and the mitochondria state under ischemia suggest an insufficient energy supply in the cortex cells. Since the brain consists of different cell types e.g. neurons and glial cells, it will be interesting to evaluate which type of brain cell mainly contributes to mitochondrial dysfunction following ischemic insult. However, with the lack of efficient methods to separate neuronal and glial cells from the tissue homogenates, new techniques will be needed for achieving this goal.

In brain tissues, amino acids not only work as energy-providing materials, many, including GABA, glycine, etc., also serve as functional neurotransmitters. In our study, upon ischemia/reperfusion, GABA and glycine were up regulated in the hippocampus and/or cortex tissues of female mice, and these observations are consistent with the previously published data by Andine et al. [24] and Melani et al. [25]. Both the releasing of GABA and the up-regulation of glycine represent the self-protective efforts of brain tissues against BCCAO-induced dysfunctions [26–28].

Other than the disturbed energy metabolism and amino acid metabolism, oxidative stress was also observed in the regional brain tissues of female BCCAO mice. Upon ischemia/reperfusion operation, the levels of three oxidative stress-related metabolites including taurine, succinate, and malonate were significantly disturbed in the cortex tissues of BCCAO female mice, while the increased level of malonate was observed in the hippocampus samples from the female experimental group (Fig. 4). Banks et al. [29] found that pretreatment of the cells with taurine could reduce oxidative stress. In the work reported by Balkan et al. [30], taurine administration could protect rats’ liver from thioacetamide-induced cirrhosis by decreasing oxidative stress. Therefore, the observed down-regulation of taurine in the cortex of female BCCAO mice suggested that oxidative stress may be induced by ischemia/reperfusion. Meanwhile, up-regulations of malonate and succinate further supported this hypothesis. Malonate has been found to be a reversible succinate dehydrogenase (SDH) inhibitor. SDH inhibition was reported to serve as a reactive oxygen species (ROS) sensor for ROS/oxidative stress [31, 32]. Therefore, inhibition of the activity of SDH by malonate may lead to accumulation of succinate in the cortex of female BCCAO mice.

Finally, although multiple protecting mechanisms, including up-regulation of protein break-down and inhibitory neurotransmitter emission, were initiated, neuronal dysfunction of female BCCAO mice still occurred. The levels of cell-state indicators, including NAA, myo-inositol, and o-phosphocholine, in the cortex tissues of female BCCAO mice were significantly perturbed, and in the hippocampus tissues of female BCCAO mice, the level of o-phosphocholine was significantly down regulated (Fig. 4). NAA, which predominantly presents in neuronal cells and acts as a neuronal marker [33, 34], was down regulated in the cortex of female BCCAO mice in the present study. This observation indicated that significant neuronal dysfunction happened in the cortex region. Another cell-state indicator, o-phosphocholine, was also found to be down regulated in the cortex and hippocampus tissues of female BCCAO mice. Since o-phosphocholine is an important component of cell membrane phospholipids, its decreased level suggested that cell membrane dysfunction was induced by ischemia/reperfusion in these two brain regions. Myo-inositol is one of the important cellular osmolytes. The increased level of myo-inositol in the cortex tissues of female BCCAO mice indicated local osmolality disorders [35], which intimately related to the decrease of the neuronal cell vitality.

### Potential biochemical mechanisms related to ischemia-induced cerebral disorders of male mice

Compared with the female experimental mice, after ischemia/reperfusion operation, similar metabolic profile change patterns were observed in the regional brain tissues of the BCCAO male mice (Fig. 4). The up-regulations of tyrosine, leucine, lysine, valine, isoleucine, glutamine and the down-regulations of glutamate and aspartate in the cortex and/or hippocampus of male BCCAO mice were detected. These results indicated that the energy metabolism disorders occurred upon global ischemia. However, no significant change was detected in AMP, which indicated that the total energy supply in regional brain tissues of male mice have not been significantly affected by BCCAO procedure. For the functional amino acids including GABA and glycine, their levels increased in the cortex of male BCCAO mice, indicating that ischemia/reperfusion also triggered similar inhibitory neurotransmitter activation to which occurred in the cortex of female BCCAO mice (Fig. 4). Compared with the female BCCAO mice, a similar oxidative stress was also detected in the cortex and hippocampus tissues of male BCCAO mice. However, it is worth noting that, although sharing a similar metabolic profile change pattern with the BCCAO female mice, the cortex region of male BCCAO mice had fewer metabolites (AMP, isobutyrate, DMA, malonate, and myo-inositol) perturbed by the cerebral ischemia/reperfusion (Additional file 1: Tables S2, S3; Fig. 4). And these data suggest that the ischemia-induced metabolic dysfunctions in the cortex of female BCCAO mice were more severe than in the male ones.

## Conclusions

In conclusion, during the early stages of global cerebral ischemia, multiple amino acids were the dominantly affected metabolites in brain tissues of the experimental mice, suggesting that the changes of amino acid metabolic pathways play key roles in the early stage of cerebral ischemia-related diseases. In addition, the change patterns of the cerebral metabolic profile induced by the global ischemia were almost the same in the female as in the male mice, as reflected by the well-matched identified perturbed metabolites in these two gender groups. However, the extent of dysfunctions that occurred in the cortex tissues of female ischemic mice were more severe than that which occurred in the cortex tissues of male ischemic mice. Fewer metabolites were disturbed by the BCCAO operation in the cortex of male mice, with AMP level only significantly affected in regional brain tissues of female BCCAO mice, but not in the male ones. Overall, for the first time, our study verified the different impact of early stage global ischemia on cerebral metabolites in male and female mice, which may shed some light on the progression of global ischemia-associated diseases and provide an important hypothesis for a better understanding of the clinically observed gender-dependent pathological outcomes in cerebral ischemia.

## Methods

### Reagents and materials

NaH_2_PO_4_∙2H_2_O and Na_2_HPO_4_∙12H_2_O (all in analytical grade) were provided by Sinopharm Chemical Reagent Co. Ltd. (Shanghai, China). D_2_O (99.9 % in D) was obtained from Sigma Chemical Corp. (St. Louis, MO, USA).

### Animal cerebral ischemia/reperfusion injury induced by BCCAO operation

ICR strain mice (8–10 weeks, SPF degree, 22–25 g in weight), were supplied by the Shanghai Experimental Animal Center, Chinese Academy of Sciences (Shanghai, China). All animals were housed in colony cages on a 12/12-h light/dark cycle at an ambient temperature of 22–25 °C and 50–60 % relative humidity. They were provided with a certified standard diet and tap water ad libitum during the experiments.

A total number of 24 mice prepared for NMR study were randomly divided into four groups with six mice in each group: the female sham group, the female experimental group, the male sham group and the male experimental group. Additional mice were randomly divided into four groups with 6–8 mice in each group for measurement of brain mitochondria swelling: the female sham group, the female experimental group, the male sham group, and the male experimental group. All the mice in experimental groups were then subjected to the surgical procedure of bilateral common carotid artery occlusion and reperfusion by following the protocol reported previously [36–38]. Briefly, at the beginning of the procedure, the mice were anesthetized with 10 % chloral hydrate by intraperitoneal injection (3.5 ml/kg). Then, an incision was made in the middle of mouse neck, and the bilateral common carotid arteries were isolated. Both arteries were then occluded twice with microvascular clips for 10 min each time. Right after the first occlusion procedure, a 15-min reperfusion was applied (ischemia for 10 min—reperfusion for 15 min—ischemia for 10 min). The mice were kept warm (body temperature of 35 °C) during the whole surgical procedure. After the procedure, each mouse was further supplemented with 1 mL normal saline (NS) by intraperitoneal injection. For those mice in the sham groups, a sham procedure was performed following the same surgical procedure described above but without artery occlusion and normal saline supplementation. Of note, due to the poor scan quality of original NMR spectra caused by limited quantities of mouse hippocampus tissues obtained, one hippocampus sample in the sham male group was excluded from the analysis.

### Preparation of brain mitochondria

Brain mitochondria were isolated from male and female ICR mice in experimental and sham groups according to previously reported methods [39, 40] with brief modification. Each mouse was decapitated and the whole cortex from both hemispheres was rapidly removed, washed, minced, and homogenized in ice-cold isolation buffer (320 mM sucrose, 10 mM Tris–HCl, 1 mM EDTA, 0.1 % BSA, pH 7.4) with Dounce homogenizer. The homogenate was centrifuged at 1000 rpm for 5 min at 4 °C. The supernatant was collected and subjected to another centrifugation at 1000 rpm for 5 min at 4 °C, and then the supernatant was centrifuged at 10,000 rpm for 10 min at 4 °C. The final mitochondrial pellet was resuspended in an ice-cold resuspension buffer and protein concentrations were adjusted to 0.6 mg/mL. The mitochondrial samples were then ready for measurement of mitochondrial swelling.

### Analysis of mitochondrial swelling

Mitochondrial swelling was measured by following a previously described protocol [41]. Briefly, after being initiated by 250 mM calcium chloride and 5 mM sodium succinate, the osmotic volume changes of mitochondria were estimated by changes in light scattering at 540 nm (30 °C) as monitored continuously for 30 cycles (the interval between trials was 30 s) with a microplate reader (Envision, Perkin Elmer). A decrease in absorbance indicates an increase in mitochondrial swelling. The decreased slope of the absorbance at 540 nm from cycle 1 to cycle 30 was calculated with linear regression to represent mitochondrial swelling degree.

### NMR sample preparation

One hour after the surgery, all of the experimental mice were sacrificed by decapitation. The brain tissue samples from the left hemisphere of the cortex and hippocampus were then quickly removed from each mouse, placed in the hard tissue-homogenizing tube, snap-frozen in liquid nitrogen and subsequently stored at −80 °C before NMR analysis.

Lyophilized aqueous brain extracts were prepared using the methanol/chloroform/water system as previously reported [12, 42]. The mixtures were allowed to thaw for 3 min, and then followed by 2 × 20 s beating of 5000 rpm with a 20 s pause between the bead beatings using a tissue homogenizer (precellys 24, Bertin technologies, Villeurbanne, France). After a 5-min incubation at 4 °C, the extracted samples were centrifuged at 12,000 rpm for 10 min at 4 °C. The upper aqueous phase was transferred into a marked 15 mL centrifugation tube and lyophilized. The powder of the extract was dissolved in 550 μL of phosphate buffer (Na_2_HPO_4_/NaH_2_PO_4_, 0.2 M, pH 7.4) and vortexed. After a centrifugation (11,000 rpm) at 4 °C for 10 min had been done, aliquots of the supernatants (500 μL) were transferred into 5-mm NMR tubes for NMR data acquisition.

## ^1^H NMR data acquisition

All NMR spectra were recorded at 298 K on a Bruker Avance III 600 NMR spectrometer (Bruker Biospin, Germany) equipped with a cryogenic probe operating at 600 MHz for ^1^H resonance. NMR data for each sample was recorded using a solvent-suppressed 1D ^1^H NOESY (NoesyPr1d) pulse sequence (RD-90°-t_1_-90°-t_m_-90°-ACQ). For the spectra of cortex samples, four dummy scans and 256 free induction decays (FIDs) were collected into 64 K data points, with a spectral width of 9578.54 Hz. For the spectra of hippocampus samples, four dummy scans and 336 FIDs were collected into 64 K data points, with a spectral width of 9578.54 Hz.

### Multivariate statistical analysis

Processed ^1^H NMR spectra were referenced to the methyl group of creatine at δ 3.043 and manually aligned using the software of MestReNova (Version 8.0, Mestrelab Research SL). For the spectra of cortex samples, the spectral region ranging from δ 0.712 to δ 9.367 was divided into 2886 integral bins with a bin width of 0.003 ppm. Moreover, the spectral regions ranging from δ 3.319 to δ 3.392 and from δ 4.678 to δ 5.230 were removed from the analysis to eliminate the effects of signals from residual methanol and imperfect water suppression. For the spectra of hippocampus samples, the spectral region ranging from δ 0.721 to δ 9.381 was bucketed into 2888 integral bins with a bin width of 0.003 ppm. The spectral regions ranging from δ 3.321 to δ 3.391 and from δ 4.540 to δ 5.230 were eliminated from the analysis due to the same reasons as those mentioned above.

The integrals of resulting metabolites were normalized to the sum of spectral intensities of all identified metabolites to compensate for the differences in the concentrations of samples. Subsequently, the integral values were imported to SIMCA-P + 12.0 software package (Umetrics, Umeå, Sweden) for PCA, PLS-DA and OPLS-DA analysis with unit variance (UV) scaling. The PCA and PLS-DA score plots were visualized with the first principal component (t[1]) and the second principal component (t[2]), while OPLS-DA plots were calculated with the first principal component (t[1]) and the orthogonal component (to[1]). The parameters Q2 (cum) and R2X (cum) were calculated to test the validity of the model against overfitting. The six-fold cross-validation method and permutation test for 350 times with the first component were carried out to measure the robustness of the PLS-DA model. The correlation coefficients of the variables relative to the first predictive component in the OPLS-DA model were extracted from S-plot. Cutoff values with significant level of 0.05 were applied to identify variables that were responsible for the discriminations of the groups [43]. The values of variable importance in the projection (VIP) were also used to evaluate the differentiating metabolites with the VIP value >1, which contributed significantly to the group clustering. The OPLS-DA loading plots, in which the VIP and r values of metabolites are displayed, were used to illustrate the relative metabolite changes induced by ischemia.

Group means of metabolites’ integrals were expressed as the mean ± std (standard deviation) and the average changes of metabolites between the experimental group and sham group were calculated (Additional file 1: Tables S2, S3) [44]. Significant differences in the mean values were evaluated by Student’s *t* test and multivariate statistical analysis. Statistical significance was considered at two or more requirements among “VIP > 1, |r| > the cutoff value and *p* < 0.05” obeying the boundary conditions.

## Additional file

10.1186/s12868-016-0256-9 The 600-MHz ^1^H NMR NOESY spectra (δ 0.9-4.7, 5.3-9.4) of aqueous extracts from the hippocampus tissues of mice in female model group (A) and female sham group (B). The abbreviations of metabolites were denoted in Table S1. **Figure S2.** The PCA score plots, PLS-DA score plots and their corresponding validation plots derived from the 600 MHz ^1^H NMR spectra of cortex samples (A, A’, A’’) and hippocampus samples (B, B’, B’’) extracted from the mice in female sham group (■) and female model group (▲). The validation plots were obtained by using a permutation test that was randomly permuted for 350 times with the first component extracts. ▲ is for R2Y (cum), and ■ is for Q2 (cum). The vertical axis of validation plot represents the R2 and Q2 values, and the horizontal axis (A’’, B’’) represents the correlation coefficients. **Figure S3.** The PCA score plots, PLS-DA score plots and their corresponding validation plots derived from the 600 MHz ^1^H NMR spectra of cortex samples (A, A’, A’’) and hippocampus samples (B, B’, B’’) extracted from the mice in male sham group (■) and male model group (▲). The validation plots were obtained by using a permutation test that was randomly permuted for 350 times with the first component extracts. ▲ is for R2Y (cum), and ■ is for Q2 (cum). The vertical axis of validation plot represents the R2 and Q2 values, and the horizontal axis (A’’, B’’) represents the correlation coefficients. Of note, due to the poor scan qualities of original NMR spectra caused by the limited quantities of mouse hippocampus tissues, one hippocampus sample in the sham male group was excluded from the analysis. **Figure S4.** Influence of BCCAO on mitochondrial swelling. (A). The Ca^2+^ induced mitochondria swelling in the cerebral cortex tissues of mice in female/male model versus sham group. **P* < 0.05, ****P* < 0.005 versus sham group. (B). Representative swelling curves of mitochondria isolated from sham or BCCAO groups (Data are expressed as mean value of each group). **Figure S5.** The 600-MHz ^1^H NMR NOESY spectra of aqueous extracts with a total number of 11 samples from the hippocampus tissues of mice in sham groups. **Figure S6.** The 600-MHz ^1^H NMR NOESY spectra of aqueous extracts with a total number of 12 samples from the hippocampus tissues of mice in model groups. **Table S1.** NMR Resonance assignments of 26 aqueous metabolites extracted from regional brain tissues of experimental mice. **Table S2.** Quantitative comparisons of aqueous metabolites extracted from cerebral cortex and hippocampus tissues of female mice. **Table S3.** Quantitative comparisons of aqueous metabolites extracted from cerebral cortex and hippocampus tissues of male mice

## Abbreviations

BCCAO: bilateral common carotid arteries occlusion
BBB: blood brain barrier
NMR: nuclear magnetic resonance
PCA: principal component analysis
PLS-DA: partial least squares discriminant analysis
OPLS-DA: orthogonal partial least squares discriminant analysis
HMDB: Human Metabolome Database
GABA: γ-aminobutryric acid
GPC: sn-glycero-3-phophocholine
AMP: adenosine monophosphate
DMA: dimethylamine
NAA: *N*-acetylaspartate
TCA cycle: tricarboxylic acid cycle
SDH: succinate dehydrogenase
ROS: reactive oxygen species
NS: normal saline
NoesyPr1d: 1D ^1^H NOESY with presaturation
FIDs: free induction decays
UV: unit variance
VIP: variable importance in the projection
std: standard deviation
