# Supplementary material for: Metabolomic investigation of regional brain tissue dysfunctions induced by global cerebral ischemia
Source: BMC Neurosci. 2016 May 20;17:25. doi: 10.1186/s12868-016-0256-9 (PMC4875627; doi:10.1186/s12868-016-0256-9)
Supplement: Supplementary file 1 — 10.1186/s12868-016-0256-9 The 600-MHz 1H NMR NOESY spectra (δ 0.9-4.7, 5.3-9.4) of aqueous extracts from the hippocampus tissues of mice in female model group (A) and female sham group (B). The abbreviations of metabolites were denoted in Table S1. Figure S2. The PCA score plots, PLS-DA score plots and their corresponding validation plots derived from the 600 MHz 1H NMR spectra of cortex samples (A, A’, A’’) and hippocampus samples (B, B’, B’’) extracted from the mice in female sham group (■) and female model group (▲). The validation plots were obtained by using a permutation test that was randomly permuted for 350 times with the first component extracts. ▲ is for R2Y (cum), and ■ is for Q2 (cum). The vertical axis of validation plot represents the R2 and Q2 values, and the horizontal axis (A’’, B’’) represents the correlation coefficients. Figure S3. The PCA score plots, PLS-DA score plots and their corresponding validation plots derived from the 600 MHz 1H NMR spectra of cortex samples (A, A’, A’’) and hippocampus samples (B, B’, B’’) extracted from the mice in male sham group (■) and male model group (▲). The validation plots were obtained by using a permutation test that was randomly permuted for 350 times with the first component extracts. ▲ is for R2Y (cum), and ■ is for Q2 (cum). The vertical axis of validation plot represents the R2 and Q2 values, and the horizontal axis (A’’, B’’) represents the correlation coefficients. Of note, due to the poor scan qualities of original NMR spectra caused by the limited quantities of mouse hippocampus tissues, one hippocampus sample in the sham male group was excluded from the analysis. Figure S4. Influence of BCCAO on mitochondrial swelling. (A). The Ca2+ induced mitochondria swelling in the cerebral cortex tissues of mice in female/male model versus sham group. *P < 0.05, ***P < 0.005 versus sham group. (B). Representative swelling curves of mitochondria isolated from sham or BCCAO groups (Data are expressed as mean va [file 12868_2016_256_MOESM1_ESM.doc]

**Supplementary information**

**Supplementary Figures**

**
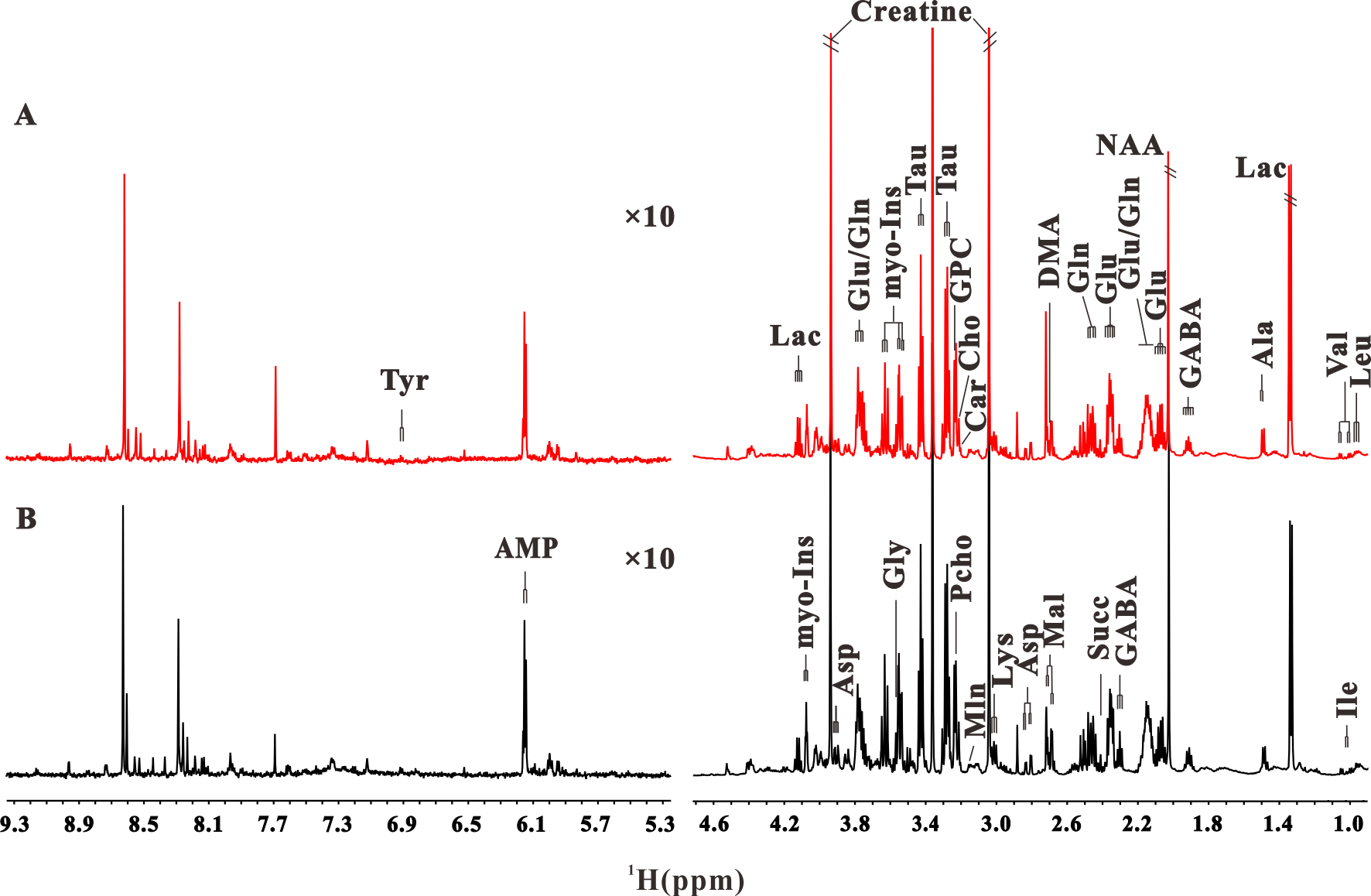
**

**Figure S1**. The 600-MHz ^1^H NMR NOESY spectra (δ 0.9-4.7, 5.3-9.4) of aqueous extracts from the hippocampus tissues of mice in female model group (A) and female sham group (B). The abbreviations of metabolites were denoted in Table S1.


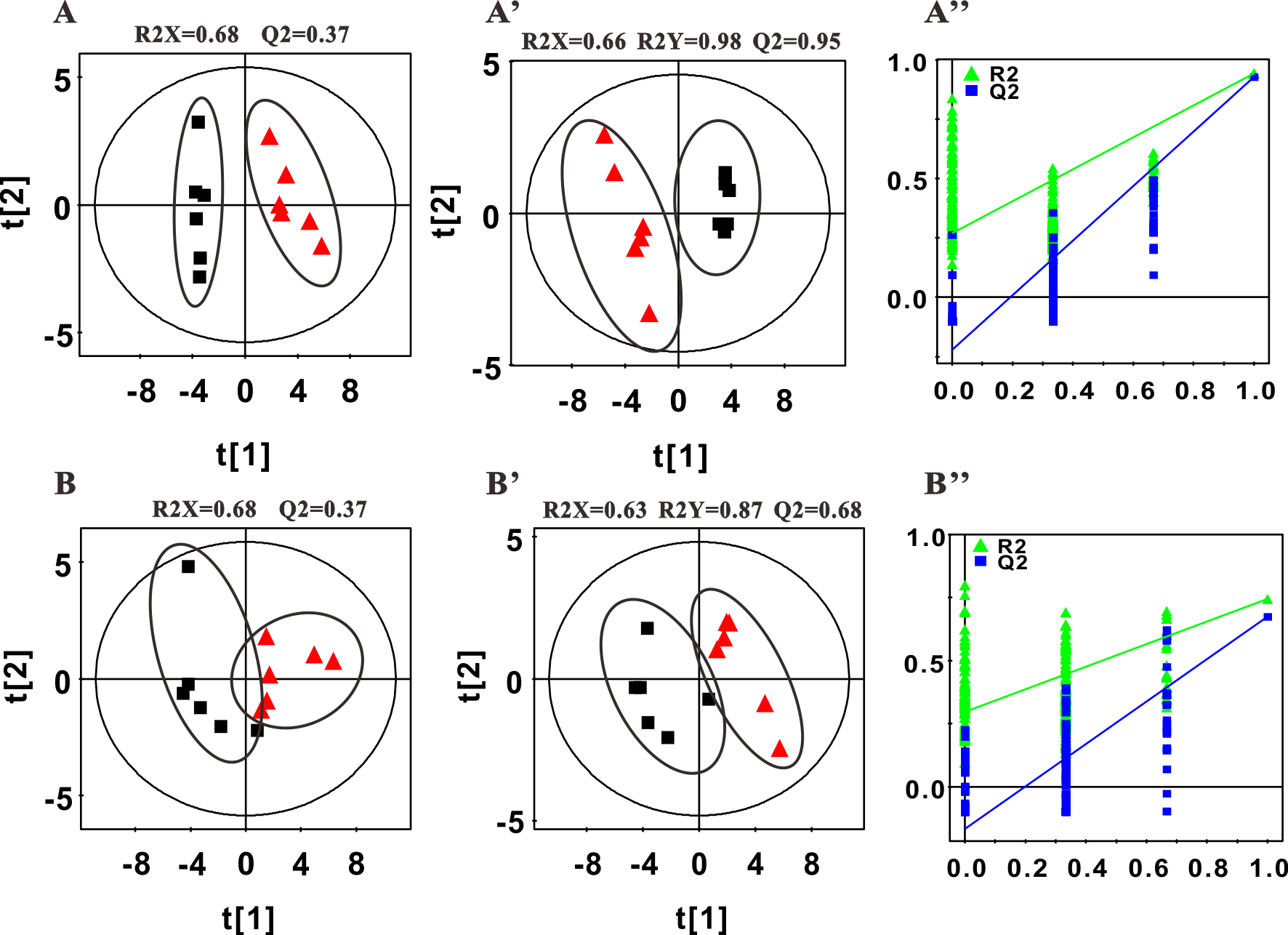


**Figure S2.** The PCA score plots, PLS-DA score plots and their corresponding validation plots derived from the 600 MHz ^1^H NMR spectra of cortex samples (A, A’, A’’) and hippocampus samples (B, B’, B’’) extracted from the mice in female sham group (■) and female model group (▲). The validation plots were obtained by using a permutation test that was randomly permuted for 350 times with the first component extracts. ▲ is for R2Y (cum), and ■ is for Q2 (cum). The vertical axis of validation plot represents the R2 and Q2 values, and the horizontal axis (A’’, B’’) represents the correlation coefficients.


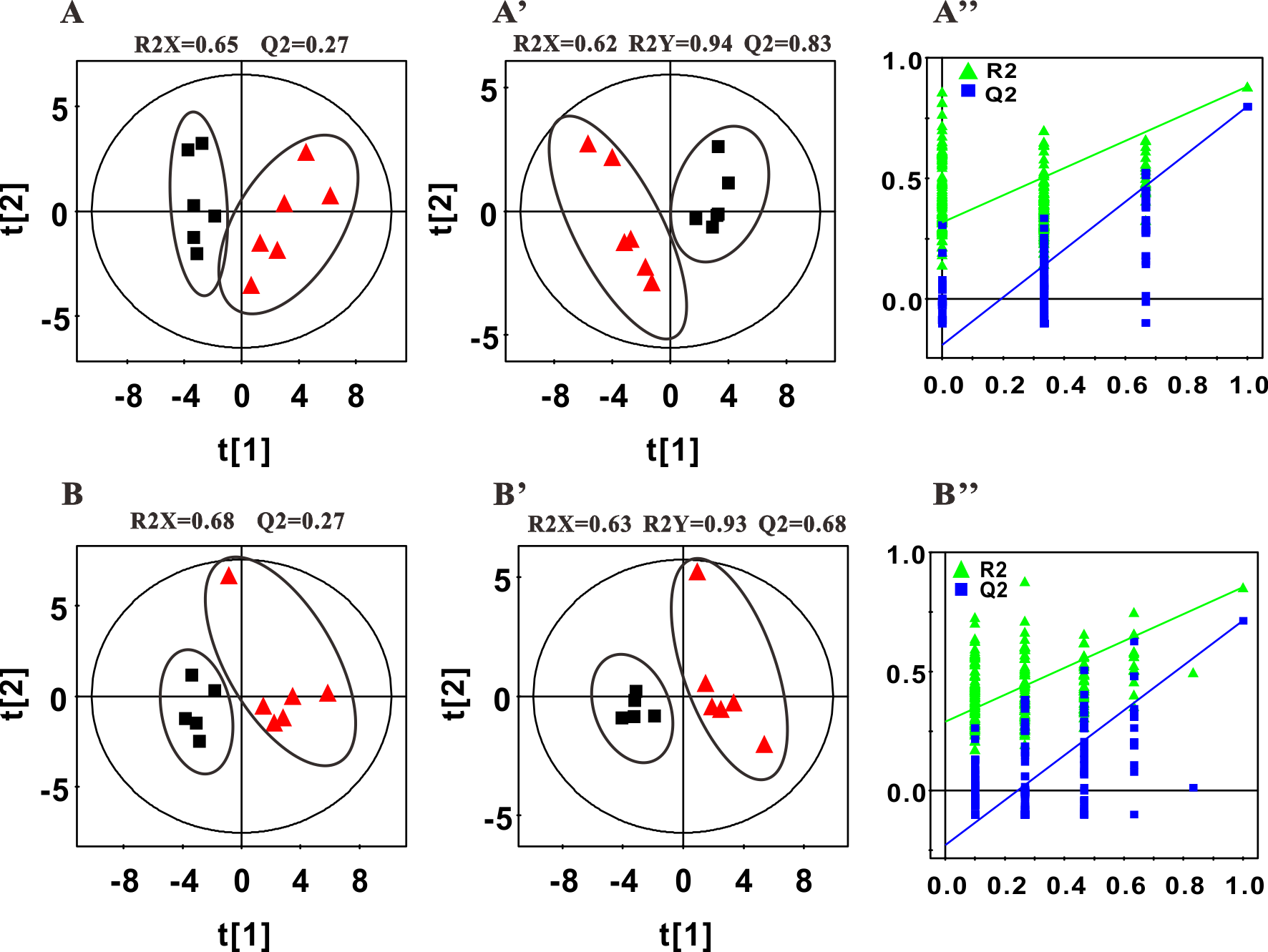


**Figure S3.** The PCA score plots, PLS-DA score plots and their corresponding validation plots derived from the 600 MHz ^1^H NMR spectra of cortex samples (A, A’, A’’) and hippocampus samples (B, B’, B’’) extracted from the mice in male sham group (■) and male model group (▲). The validation plots were obtained by using a permutation test that was randomly permuted for 350 times with the first component extracts. ▲ is for R2Y (cum), and ■ is for Q2 (cum). The vertical axis of validation plot represents the R2 and Q2 values, and the horizontal axis (A’’, B’’) represents the correlation coefficients. Of note, due to the poor scan qualities of original NMR spectra caused by the limited quantities of mouse hippocampus tissues, one hippocampus sample in the sham male group was excluded from the analysis.


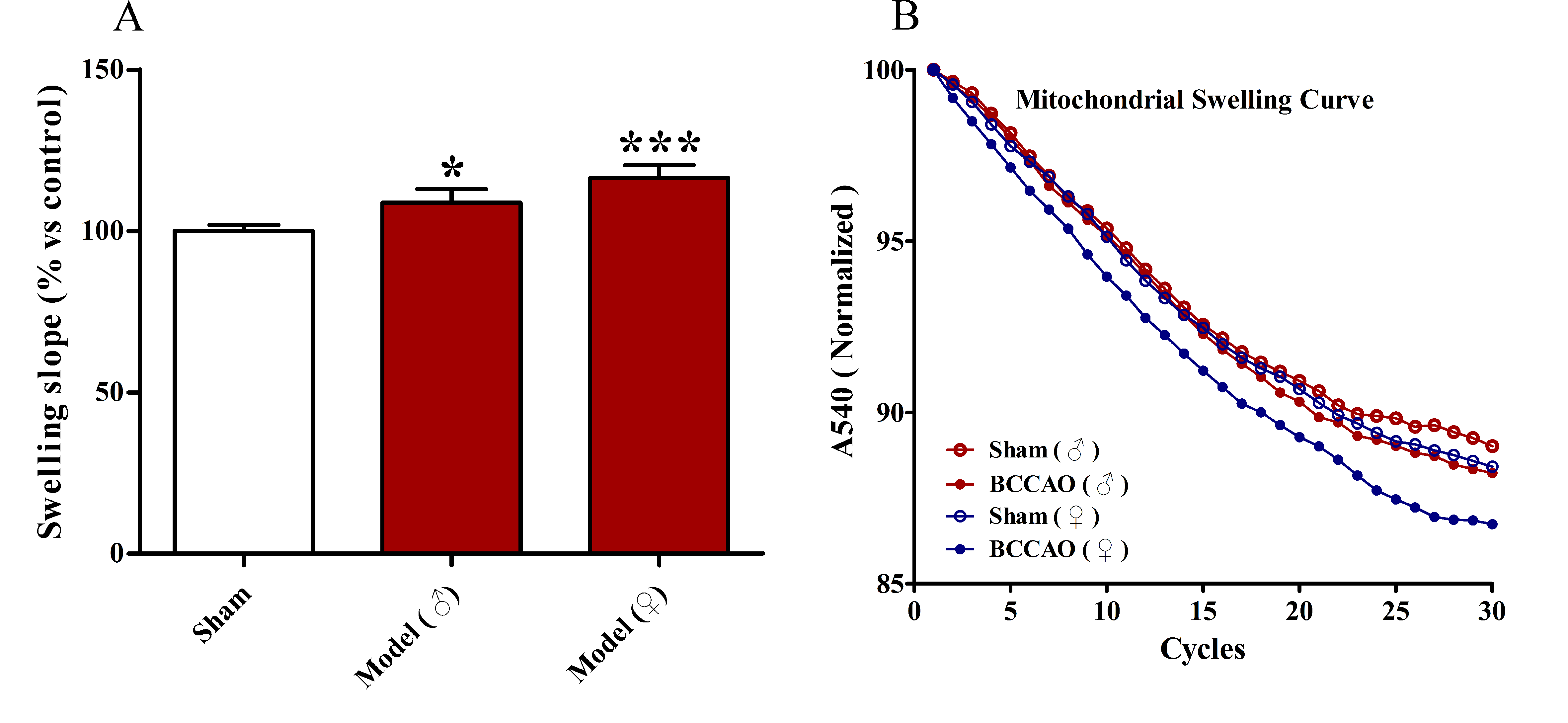


**Figure S4.** Influence of BCCAO on mitochondrial swelling. (A). The Ca^2+^ induced mitochondria swelling in the cerebral cortex tissues of mice in female/male model versus sham group. **P* < 0.05, ****P* < 0.005 versus sham group. (B). Representative swelling curves of mitochondria isolated from sham or BCCAO groups (Data are expressed as mean value of each group).

**
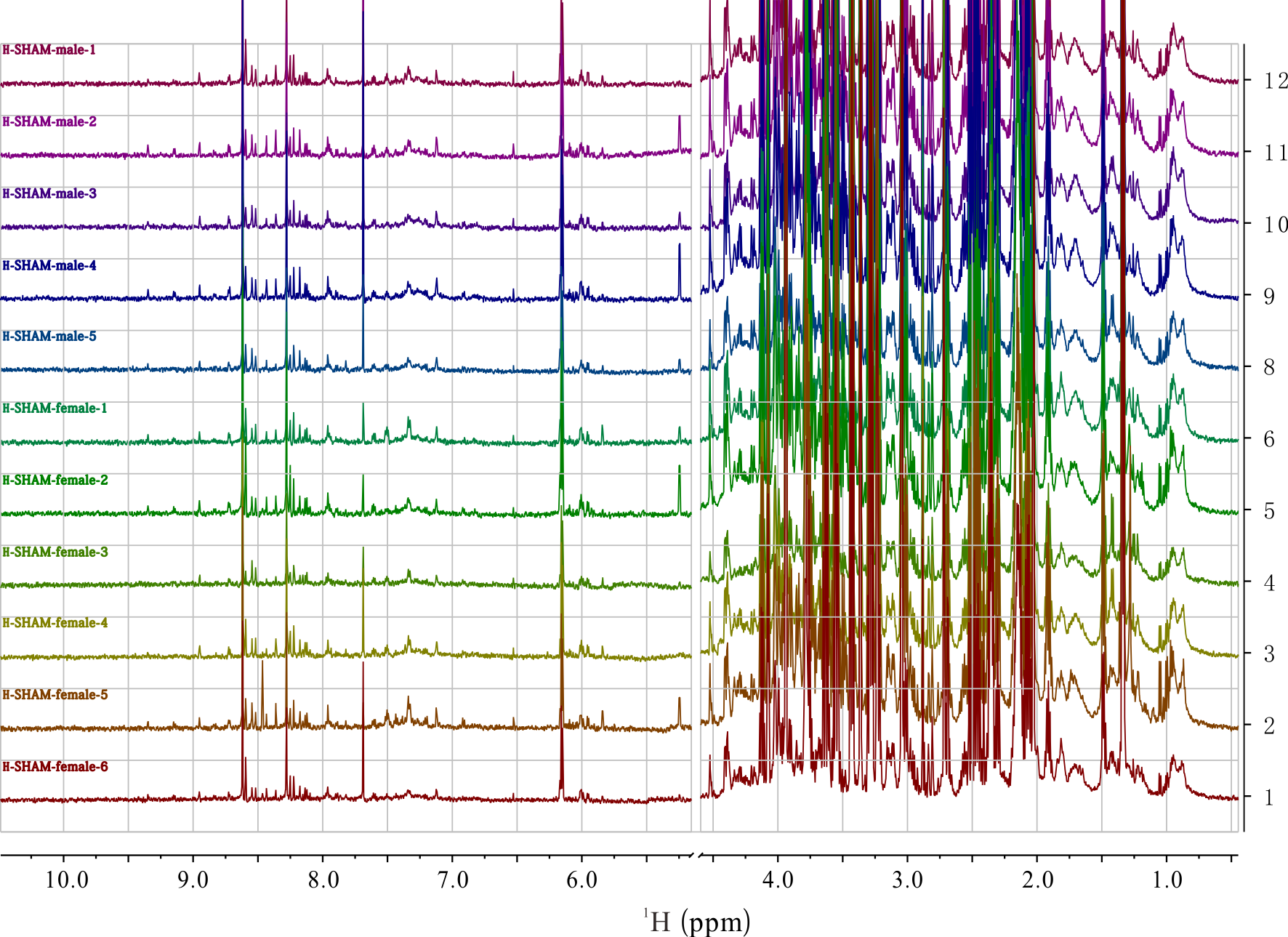
**

**Figure S5.** The 600-MHz ^1^H NMR NOESY spectra of aqueous extracts with a total number of 11 samples from the hippocampus tissues of mice in sham groups.

**
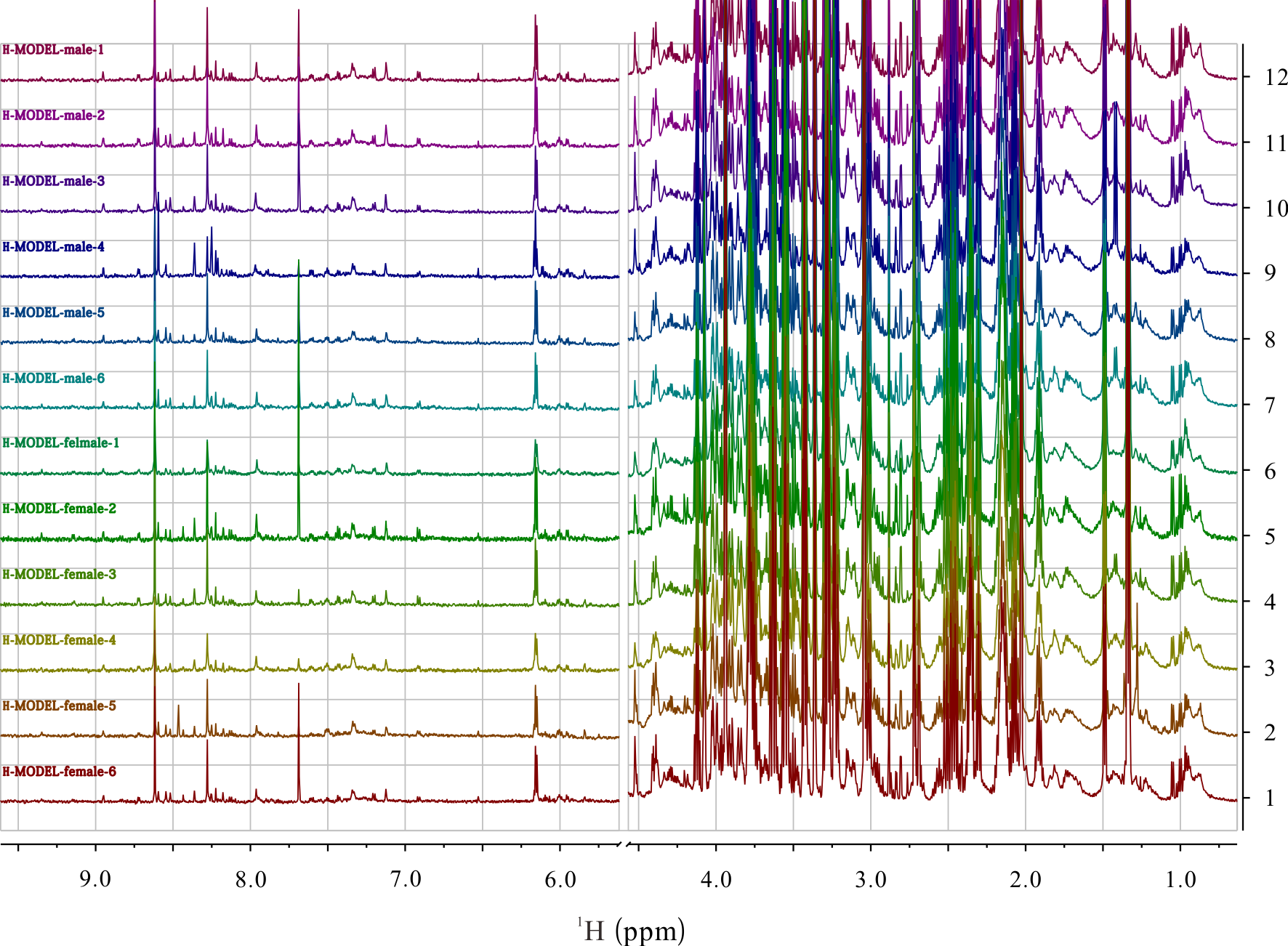
**

**Figure S6.** The 600-MHz ^1^H NMR NOESY spectra of aqueous extracts with a total number of 12 samples from the hippocampus tissues of mice in model groups.

**Supplementary Tables**

**Table S1.** NMR Resonance assignments of 26 aqueous metabolites extracted from regional brain tissues of experimental mice.

| **Metabolites(abbreviation)** | **Groups** | **δ ^1^H (multiplicity)^#^** |
| --- | --- | --- |
| **leucine (Leu)** | δ-CH_3_  δ'-CH_3_  γ-CH, β-CH_2_  α-CH | 0.96(d)  0.97(d)  1.68(m), 1.70(m),1.74(m)  3.74(m) |
| **isoleucine (Ile)** | γ-CH_3_  δ-CH_3_  half γ-CH_2_, half γ-CH_2_  β-CH  α-CH | 0.94(t)  1.01(d)  1.20 (m), 1.47(m)  1.95(m)  3.68(d) |
| **valine (Val)** | γ'-CH_3_  γ-CH_3_  β-CH  α-CH | 0.99(d)  1.05(d)  2.27(m)  3.61(d) |
| **isobutyrate (IB)** | 2×β-CH_3_  α-CH | 1.07(d)  2.49(m) |
| **lactate (Lac)** | β-CH_3_  α-CH | 1.33(d)  4.12(q) |
| **alanine (Ala)** | β-CH_3_  α-CH | 1.48(d)  3.77(q) |
| **lysine (Lys)** | ε-CH_2_  δ-CH_2_  half γ-CH_2_, half γ-CH_2_  β-CH_2_  α-CH | 3.02(t)  1.74(m)  1.50(m), 1.44(m)  1.90(m)  3.75(t) |
| **γ-aminobutryric acid**  **(GABA)** | γ-CH_2_  β-CH_2_  α-CH_2_ | 3.00(t)  1.91(m)  2.31(t) |
| **glutamate (Glu)** | γ-CH_2_  half β-CH_2_, half β-CH_2_  α-CH | 2.36(m)  2.13(m), 2.07(m)  3.77(dd) |
| **succinate (Succ)** | 2×α-CH_2_ | 2.41(s) |
| **glutamine (Gln)** | γ-CH_2_  β-CH_2_  α-CH | 2.46(m)  2.14(m)  3.79(m) |
| **aspartate (Asp)** | half β-CH_2_, half β-CH_2_  α-CH | 2.80(dd), 3.68(dd)  3.91(dd) |
| **malate (Mal)** | half β-CH_2_, half β-CH_2_  α-CH | 2.37(dd), 2.69(dd)  4.29(dd) |
| **dimethylamine (DMA)** | N-CH_3_ | 2.71(s) |
| **creatine (Cr)** | N-CH_3_  α-CH_2_ | 3.04(s)  3.94(s) |
| **malonate (Mln)** | α-CH_2_ | 3.16(s) |
| **carnitine (Car)** | N-(CH_3_)_3_  N-CH_2_  β-CH  α-CH_2_ | 3.21(s)  3.43(m)  4.58(m)  2.44(dd) |
| **choline (Cho)** | N-(CH_3_)_3_  N-CH_2_  α-CH_2_ | 3.21(s)  3.54(t)  4.06(t) |
| **o-phosphocholine (Pcho)** | N-(CH_3_)_3_  N-CH_2_  α-CH_2_ | 3.23(s)  3.60(t)  4.18(m) |
| **sn-glycero-3-phosphocholine**  **(GPC)** | N-(CH_3_)_3_  N-CH_2_  α-CH_2_  glycerol:  half γ-CH_2_, half γ-CH_2_  β-CH  half α-CH_2_, half α-CH_2_ | 3.24(s)  3.67(m)  4.32(m)  3.68(dd), 3.60(dd)  3.90(m)  3.95(m), 3.88(m) |
| **taurine (Tau)** | N-CH_2_  S-CH_2_ | 3.28(t)  3.43(t) |
| **myo-inositol (myo-Ins)** | δ-CH  2×γ-CH  2×β-CH  α-CH | 4.08(t)  3.54(dd)  3.63(t)  3.29(t) |
| **glycine (Gly)** | N-CH_2_ | 3.57(s) |
| **tyrosine (Tyr)** | phenyl moiety: 2×γ-CH, 2×β-CH.  half β-CH_2_, half β-CH_2_ | 7.17(d), 6.88(d).  3.18(dd), 3.03(dd) |
| ***N*-acetylaspartate (NAA)** | acetyl: α-CH_2_ | 2.03(s) |
| **adenosine monophosphate**  **（AMP）** | adenine moiety:  N-CH  CH  CH’ | 6.15(d)  8.62(s)  8.28(s) |

Note: ^#^ s, singlet；d, doublet；t, triplet；q, quartet；m, multiplet.

**Table S2.** Quantitative comparisons of aqueous metabolites extracted from cerebral cortex and hippocampus tissues of female mice.

| **Metabolites** | **cortex** | | | **hippocampus** | | |
| --- | --- | --- | --- | --- | --- | --- |
|  | **Integrals in the sham group^a^**  **(mean±std)*10^-4^** | **Integrals in the model group^a^**  **(mean±std)*10^-4^** | **%Average changes**  **(model *vs* sham)^b^**  **(\|r\|,VIP, *p*-Value^c^)**  **(model *vs* sham)^d^**  **(\|r\|>=0.576)** | **Integrals in the sham group^a^**  **(mean±std)*10^-4^** | **Integrals in the**  **model group^a^**  **(mean±std)*10^-4^** | **%Average changes**  **(model *vs* sham)^b^**  **(\|r\|,VIP, *p*-Value^c^)**  **(model *vs* sham)^d^**  **(\|r\|>=0.576)** |
| **Leu** | 12.49±1.64 | 30.47±7.11 | 144.00(0.91,1.22,0.00) | 19.98±5.67 | 29.21±5.49 | 46.20(0.73,1.10,0.02) |
| **Ile** | 7.18±0.40 | 16.55±4.29 | 130.44(0.88,1.19,0.00) | 7.64±2.48 | 13.13±3.37 | 71.84(0.78,1.17,0.01) |
| **Val** | 19.50±3.12 | 48.66±7.12 | 149.49(0.95,1.31,0.00) | 21.38±7.00 | 40.99±1.66 | 91.75(0.95,1.48,0.00) |
| **IB** | 0.26±0.17 | 0.87±0.37 | 233.43(0.77,1.05,0.00) | / | / | / |
| **Lac** | 1339.33±98.06 | 1188.27±167.91 | -11.28(0.50,0.71,0.09) | 1178.46±117.31 | 1053.62±156.95 | -10.59(0.50,0.72,0.15) |
| **Ala** | 76.35±9.79 | 215.07±62.04 | 181.70(0.87,1.19,0.00) | 128.07±24.43 | 225.41±38.43 | 76.01(0.91,1.40,0.00) |
| **Lys** | 273.92±9.26 | 396.63±46.96 | 44.80(0.91,1.23,0.00) | 212.32±44.15 | 285.83±87.89 | 34.62(0.57,0.82,0.10) |
| **GABA** | 277.48±27.35 | 406.53±44.13 | 46.51(0.91,1.22,0.00) | 322.07±49.31 | 445.15±67.18 | 38.22(0.82,1.23,0.00) |
| **Glu** | 1576.77±71.60 | 1290.71±62.65 | -18.14(0.92,1.27, 0.00) | 1340.84±140.74 | 1111.88±51.15 | -17.08(0.90,1.25,0.00) |
| **Succ** | 40.23±4.33 | 46.80±4.37 | 16.36(0.61,0.88,0.03) | 33.71±5.32 | 31.54±5.29 | -6.42(0.24,0.36,0.50) |
| **Gln** | 868.80±65.03 | 1026.93±64.26 | 18.20(0.80,1.11,0.00) | 826.44±80.98 | 908.63±72.65 | 9.94(0.57,0.83,0.09) |
| **Asp** | 257.71±32.20 | 179.02±16.51 | -30.53(0.86,1.19,0.00) | 141.65±22.90 | 109.26±20.10 | -22.86(0.71,1.04,0.03) |
| **Mal** | 258.26±43.74 | 279.27±23.33 | 8.14(0.33,0.43,0.32) | 538.47±261.74 | 513.40±110.91 | -4.66(0.02,0.11,0.83) |
| **DMA** | 32.07±22.36 | 57.89±12.18 | 80.51(0.66,0.85,0.03) | 70.48±68.25 | 55.57±36.34 | -21.16(0.06,0.24,0.65) |
| **Cr** | 1424.36±98.62 | 1429.44±31.43 | 0.36(0.02,0.05,0.91) | 1505.79±117.63 | 1402.00±114.60 | -6.89(0.48,0.72,0.15) |
| **Mln** | 9.78±1.23 | 22.32±4.30 | 128.18(0.92,1.25,0.00) | 46.97±3.69 | 57.78±3.85 | 23.02(0.85,1.38,0.00) |
| **Car** | 6.29±0.46 | 6.82±1.62 | 8.39(0.27,0.33,0.46) | 7.14±1.24 | 8.98±2.08 | 25.77(0.55,0.83,0.09) |
| **Cho** | 33.12±12.04 | 34.05±13.22 | 2.81(0.08,0.06,0.90) | 32.06±4.76 | 31.66±3.71 | -1.27(0.02,0.09,0.87) |
| **Pcho** | 91.52±6.03 | 70.26±5.77 | -23.23(0.89,1.23,0.00) | 90.45±10.99 | 68.20±5.74 | -24.60(0.90,1.33,0.00) |
| **GPC** | 72.22±10.14 | 74.45±9.00 | 3.09(0.16,0.17,0.70) | 62.64±6.96 | 52.81±9.59 | -15.69(0.61,0.88,0.07) |
| **Tau** | 1362.02±48.82 | 1232.67±8.28 | -9.50(0.92,1.24,0.00) | 1380.68±121.92 | 1556.51±195.38 | 12.74(0.50,0.83,0.09) |
| **myo-Ins** | 698.76±65.31 | 820.10±58.42 | 17.37(0.75,1.01,0.01) | 1008.90±116.35 | 1058.46±42.49 | 4.91(0.30,0.48,0.35) |
| **Gly** | 53.52±39.38 | 68.03±33.04 | 27.10(0.18,0.29,0.51) | 138.18±16.79 | 173.67±10.69 | 25.69(0.87,1.33,0.00) |
| **Tyr** | 2.32±0.69 | 7.47±1.05 | 222.57(0.93,1.32,0.00) | 3.61±2.41 | 8.08±2.12 | 123.57(0.75,1.20,0.01) |
| **NAA** | 944.45±69.22 | 825.69±62.82 | -12.57(0.70,0.97,0.01) | 676.47±29.97 | 610.23±89.84 | -9.79(0.51,0.78,0.12) |
| **AMP** | 261.30±15.95 | 225.04±25.31 | -13.88(0.71,0.94,0.01) | 205.62±18.93 | 147.98±29.87 | -28.03(0.82,1.28,0.00) |

Note: the levels of metabolites colored in red/green increased/decreased in model group mice.

The levels of metabolites colored in black showed no significant change in model group mice.

^a^ The relative integrals of metabolites were determined from 1D ^1^H NMR analysis of brain aqueous extracts of each group mice.

^b^ Values are represented as the fold-induction of peak integral between groups.

^c^ The *p*-values were obtained from student's *t*-test.

^d^ The absolute values of correlation number extracted from the correlation plots of OPLS-DA models. The cutoff value is 0.576 in the correlation-loading plots of the cortex and hippocampus samples of model group vs sham group.

**Table S3.** Quantitative comparisons of aqueous metabolites extracted from cerebral cortex and hippocampus tissues of male mice.

| **Metabolites** | **cortex** | | | **hippocampus** | | |
| --- | --- | --- | --- | --- | --- | --- |
|  | **Integrals in the sham group^a^**  **(mean±std)*10^-4^** | **Integrals in the model group^a^**  **(mean±std)*10^-4^** | **%Average changes**  **(model *vs* sham)^b^**  **(\|r\|,VIP*, p*-Value^c^)**  **(model *vs* sham)^d^**  **(\|r\|>=0.576)** | **Integrals in the sham group^a^**  **(mean±std)*10^-4^** | **Integrals in the model group^a^**  **(mean±std)*10^-4^** | **%Average changes**  **(model *vs* sham)^b^**  **(\|r\|, VIP, *p*-Value^c^)**  **(model *vs* sham)^d^**  **(\|r\|>=0.602)** |
| **Leu** | 13.93±2.94 | 24.33±9.36 | 74.70(0.65,1.00,0.03) | 19.05±3.88 | 26.18±6.80 | 37.39(0.60,0.95,0.07) |
| **Ile** | 7.81±1.67 | 13.23±5.26 | 69.48(0.64,0.96,0.04) | 7.52±0.68 | 11.81±3.03 | 57.07(0.72,1.20,0.01) |
| **Val** | 19.19±2.37 | 42.49±12.04 | 121.38(0.84,1.31,0.00) | 17.63±1.12 | 38.03±9.54 | 115.64(0.86,1.42,0.00) |
| **IB** | 0.35±0.23 | 0.43±0.28 | 20.49(0.14,0.24,0.63) | / | / | / |
| **Lac** | 1267.66±96.62 | 1225.91±113.07 | -3.29(0.16,0.34,0.51) | 1201.19±144.99 | 1092.33±122.23 | -9.06(0.32,0.69,0.21) |
| **Ala** | 75.18±10.47 | 136.06±51.46 | 81.00(0.71,1.06,0.02) | 115.60±16.02 | 187.75±35.58 | 62.41(0.83,1.36,0.00) |
| **Lys** | 268.37±17.27 | 344.64±53.76 | 28.42(0.75,1.14,0.01) | 220.16±28.24 | 229.10±26.66 | 4.06(0.21,0.30,0.60) |
| **GABA** | 255.82±20.60 | 364.40±42.54 | 42.45(0.87,1.38,0.00) | 308.17±15.25 | 406.34±33.22 | 31.86(0.92,1.50,0.00) |
| **Glu** | 1561.92±57.61 | 1297.32±89.25 | -16.94(0.91,1.40,0.00) | 1391.17±71.53 | 1143.96±96.71 | -17.77(0.82,1.42,0.00) |
| **Succ** | 42.31±4.71 | 48.49±2.39 | 14.60(0.73,1.06,0.02) | 33.63±2.60 | 27.76±7.78 | -17.44(0.53,0.79,0.14) |
| **Gln** | 812.62±29.20 | 1064.29±77.41 | 30.97(0.92,1.45,0.00) | 765.16±40.46 | 923.20±89.04 | 20.65(0.70,1.30,0.01) |
| **Asp** | 248.44±15.07 | 196.72±21.51 | -20.82(0.89,1.32,0.00) | 152.21±15.45 | 125.72±24.36 | -17.41(0.66,0.96,0.07) |
| **Mal** | 261.47±24.59 | 227.47±34.64 | -13.01(0.47,0.83,0.08) | 386.47±136.29 | 527.49±111.74 | 36.49(0.59,0.89,0.09) |
| **DMA** | 27.78±24.98 | 37.71±16.79 | 35.72(0.32,0.39,0.44) | 29.76±16.44 | 71.15±47.37 | 139.07(0.65,0.88,0.10) |
| **Cr** | 1503.22±59.15 | 1476.29±42.75 | -1.79(0.37,0.43,0.39) | 1543.94±40.21 | 1456.33±100.03 | -5.67(0.63,0.87,0.10) |
| **Mln** | 11.13±0.74 | 14.22±4.46 | 27.74(0.51,0.74,0.13) | 47.85±2.75 | 56.51±5.35 | 18.09(0.64,1.23,0.01) |
| **Car** | 5.95±0.48 | 5.83±0.60 | -1.97(0.18,0.19,0.71) | 8.51±1.35 | 8.07±1.89 | -5.20(0.32,0.24,0.67) |
| **Cho** | 24.76±4.90 | 23.48±3.58 | -5.13(0.20,0.25,0.62) | 35.48±7.62 | 45.95±26.15 | 29.52(0.33,0.46,0.41) |
| **Pcho** | 95.11±6.73 | 70.84±4.43 | -25.51(0.91,1.45,0.00) | 104.66±11.14 | 82.57±18.72 | -21.10(0.63,1.02,0.05) |
| **GPC** | 72.02±4.67 | 69.45±7.21 | -3.57(0.17,0.36,0.48) | 63.65±5.44 | 53.84±5.42 | -15.41(0.78,1.18,0.02) |
| **Tau** | 1435.06±27.39 | 1312.65±88.84 | -8.53(0.74,1.13,0.01) | 1576.21±121.12 | 1514.52±89.05 | -3.91(0.46,0.52,0.36) |
| **myo-Ins** | 773.46±69.31 | 776.08±62.67 | 0.34(0.02,0.03,0.95) | 976.10±61.53 | 960.36±81.57 | -1.61(0.06,0.20,0.73) |
| **Gly** | 62.25±9.32 | 99.18±8.84 | 59.33(0.96,1.44,0.00) | 134.62±13.13 | 160.40±3.42 | 19.15(0.88,1.41,0.00) |
| **Tyr** | 3.01±0.71 | 7.79±1.26 | 159.03(0.95,1.47,0.00) | 4.43±2.24 | 8.42±3.71 | 90.20(0.60,0.96,0.07) |
| **NAA** | 904.84±13.06 | 863.67±30.81 | -4.55(0.73,1.09,0.01) | 663.45±26.59 | 673.98±50.50 | 1.59(0.15,0.23,0.69) |
| **AMP** | 246.34±20.83 | 257.05±8.45 | 4.35(0.40,0.55,0.27) | 193.39±23.68 | 168.23±17.42 | -13.01(0.65,0.94,0.07) |

Note: the levels of metabolites colored in red/green increased/decreased in model group mice.

The levels of metabolites colored in black showed no significant change in model group mice.

^a^ The relative integrals of metabolites were determined from 1D ^1^H NMR analysis of brain aqueous extracts of each group mice.

^b^ Values are represented as the fold-induction of peak integral between groups.

^c^ The *p*-values were obtained from student's *t*-test.

^d^ The absolute values of correlation number extracted from the correlation plots of OPLS-DA models. The cutoff values for the correlation-loading plots of the cortex and hippocampus samples of model group vs sham group are 0.576 and 0.602, respectively.
